# Improvement of Reliability Determination Performance of Real Time Kinematic Solutions Using Height Trajectory

**DOI:** 10.3390/s21020657

**Published:** 2021-01-19

**Authors:** Aoki Takanose, Yoshiki Atsumi, Kanamu Takikawa, Junichi Meguro

**Affiliations:** Department of Mechatronics Engineering, Faculty of Science and Technology, Meijo University, Nagoya 468-8502, Japan; 203432001@ccmailg.meijo-u.ac.jp (Y.A.); 193432010@ccmailg.meijo-u.ac.jp (K.T.); meguro@meijo-u.ac.jp (J.M.)

**Keywords:** global navigation satellite system (GNSS), real time kinematic (RTK), position reliability estimation, ratio-test, urban areas, multipath, height trajectory

## Abstract

Autonomous driving support systems and self-driving cars require the determination of reliable vehicle positions with high accuracy. The real time kinematic (RTK) algorithm with global navigation satellite system (GNSS) is generally employed to obtain highly accurate position information. Because RTK can estimate the fix solution, which is a centimeter-level positioning solution, it is also used as an indicator of the position reliability. However, in urban areas, the degradation of the GNSS signal environment poses a challenge. Multipath noise caused by surrounding tall buildings degrades the positioning accuracy. This leads to large errors in the fix solution, which is used as a measure of reliability. We propose a novel position reliability estimation method by considering two factors; one is that GNSS errors are more likely to occur in the height than in the plane direction; the other is that the height variation of the actual vehicle travel path is small compared to the amount of movement in the horizontal directions. Based on these considerations, we proposed a method to detect a reliable fix solution by estimating the height variation during driving. To verify the effectiveness of the proposed method, an evaluation test was conducted in an urban area of Tokyo. According to the evaluation test, a reliability judgment rate of 99% was achieved in an urban environment, and a plane accuracy of less than 0.3 m in RMS was achieved. The results indicate that the accuracy of the proposed method is higher than that of the conventional fix solution, demonstratingits effectiveness.

## 1. Introduction

The global navigation satellite system (GNSS) is currently being used in a variety of applications that employ position information. Among GNSS positioning methods, real time kinematic (RTK) is one of the most accurate methods and provides cm-level position estimation in an open sky environment [1]. In recent years, the development of autonomous vehicles and self-driving support systems has progressed [2,3]. RTK is occasionally employed, because automated driving requires highly accurate position information, such as a planar error below 0.3 m. RTK is used not only for automated driving, but also for automated guided vehicle (AGV) and other logistics transport robots, position estimation using light detection and ranging (LiDAR), and simultaneous localization and mapping (SLAM) integration [4,5,6]. It is moreover employed as a reference to evaluate the position results estimated by SLAM [7,8].

Currently, RTK can be used easily in several ways. Some GNSS receivers are equipped with RTK, whereas others use the open source software RTKLIB [1], which is an RTK application that uses raw data from receiver, to estimate RTK. In other cases, RTK is performed by a software receiver using an RF recorder [9,10]. RTK uses the carrier phase transmitted from the satellite to estimate the position. The pseudo-range used in single positioning and differential GNSS (DGNSS) is calculated by measuring the propagation time of the satellite signal. Therefore, because the signals are transmitted at the speed of light, a clock error of 1 µs can result in an observation error of approximately 300 m. In contrast, the carrier wave has a wavelength of approximately 0.2 m for an L1 signal, and the resolution of the phase that can be measured is high. However, only the carrier phase can be measured, and the number of waves being transmitted is unknown. This problem is called integer ambiguity, and the solution is referred to as ambiguity resolution (AR). Various methods have been proposed for AR. The most widely used is integer least-squares (ILS). Based on ILS, there are the fast ambiguity resolution approach (FARA) [11], the least-squares ambiguity decorrelation adjustment (LAMBDA) [12], and the least-squares ambiguity search technique (LSAST) [13]. Other AR methods based on the Success Rate Criterion (SRC) have also been proposed [14,15]. By accurately solving the integer ambiguity, we can obtain a fix solution, which is a centimeter-class positioning estimation result.

To improve the robustness of GNSS, a method of integrating GNSS and the inertial measurement unit (IMU) has been proposed [16,17,18,19,20,21,22,23,24,25]. GNSS cannot estimate the position in places where the signal cannot be received, such as tunnels and under elevated buildings. In such cases, the integration of IMU enables position estimation, even in places unreachable by signals. There are two main types of GNSS/IMU integration: loose coupling [18,19], which integrates the results of each sensor, and tight coupling [20,21], which integrates the raw values of each sensor. A Bayesian filter following a normal distribution, such as the Kalman filter, is commonly used for these integrations. Because the Bayesian filter takes into account the error in the estimation, the accuracy of position estimation can be improved [22,23,24]. Moreover, the integration of RTK and IMU allows for a very accurate position estimation. In this case, the integration is based on trusting the fix solution of RTK. Therefore, when integrating RTK and IMU, the accuracy and reliability of the fix solution is highly important.

GNSS-based position estimation causes accuracy degradation in urban areas. In urban areas, where there are numerous obstructions such as high-rise buildings, the number of observation satellites decreases, and multipath occurs due to reflection and diffraction of satellite signals [26,27]. In recent years, multi-GNSS has become more common owing to the increase in the number of satellite systems [28,29,30,31]. The main multi-GNSS are the global positioning system (GPS) of the United States, BeiDou navigation satellite system (BDS) of China, global navigation satellite system (GLONASS) of Russia, and Galileo of the EU. Multi-GNSS solves the problem of reduced number of observation satellites and improves the utilization rate. In contrast, the signal multipath is directly related to the decrease in the position estimation accuracy. The integration of GNSS/IMU is considered to be effective for this problem [32,33]. However, the error due to multipath does not follow a normal distribution. In the Kalman filter, where the error is assumed to be normally distributed, it may diverge due to multipath noise. Understanding the error due to multipath is difficult. Therefore, the integration of GNSS/IMU is not effective unless the amount of error due to multipath is detected or the integration is performed by removing multipath noise [34]. One solution is to use a GNSS/IMU system; however, its use is limited due to its high cost. Furthermore, multipath degrades the accuracy of the fix solutions of RTK. In urban areas, a fix solution with a non-negligible error may be generated and this is referred to as the missed fix. In such cases, it becomes difficult to use the fix solution as a reliability indicator. Therefore, there is a risk of failure in integration that relies on the fix solution.

Therefore, we propose a novel method to determine the reliability of the fix solution. The goal is to determine whether the position error is within 0.3 m, which is required for applications such as automatic driving. The reliability of the proposed method is assessed not by the horizontal plane, but by the height direction. Two reasons justify our focus on the height direction. The first is that GNSS positioning errors in the height direction tend to be larger than those in the plane due to geometric factors between the satellite and the receiver. The second is because we can accurately estimate the vehicle motion with multipath removed. For these two reasons, we can accurately determine the fix solution even with low-cost GNSS/IMU by focusing on the height direction. If the proposed method is effective, conventional GNSS/IMU integration methods may also operate effectively in urban areas.

The paper is organized as follows. In Section 2, we explain the generation mechanism of the fix solutions with errors and conventional testing methods. Section 3 provides a detailed description of the proposed method for determining the reliability. In Section 4, we discuss the evaluation tests and results of the proposed method. Section 5 provides the summary and conclusion of the paper.

## 2. LAMBDA Method and Ratio Test

RTK is a high-precision positioning method using the carrier phase. However, its integer ambiguity is unknown. To address this problem, various methods have been devised. In this study, we describe the LAMBDA method, which is used in RTK algorithm. In RTK algorithm, the state variables are set as follows:

(1) $$x=\left(\begin{array}{c} a\\ b \end{array}\right),\;Q=\left(\begin{array}{cc} Q_{aa} & Q_{ab}\\ Q_{ba} & Q_{bb} \end{array}\right)$$

where *x* is the state vector, *a* is the integer ambiguity, *b* is the real value parameter, and *Q* is the error covariance. The observation model is defined as follows:

(2) y=Hx+v

where *y* is the observation vector, containing the pseudo-range and carrier phase, *H* is the observation matrix, and *v* is the observation noise. In RTKLIB, the first step is to solve the problem using the Kalman filter with integer ambiguities as real values. The resulting real-valued ambiguity is called the float ambiguity. Then, the float ambiguity is solved by the ambiguity resolution (AR) as an integer value. A simple AR method is the integer least squares method, which has the following evaluation function.

(3) $$C\left(a_{fix}\right)=argmin{\left(a_{fix}-a_{float}\right)}^{T}Q_{aa}{}^{-1}\left(a_{fix}-a_{float}\right)$$

When the error covariance of the float ambiguity is a true diagonal matrix, rounding off the float solution is used to obtain the optimal solution of the fix solution. However, the actual error variance is not a diagonal matrix. Because there are error correlations for each float ambiguity, estimation by search is required instead of rounding. Consequently, the search cost is high.

Therefore, the LAMBDA method transforms the variables, such that the error variance becomes a diagonal matrix. By applying the transformation and making the errors uncorrelated, it is possible to reduce the search cost for integer solutions [35,36]. For this transformation to preserve the integer nature of the ambiguity, the transformation matrix *Z* must obey the following conditions:

- *Z* is composed of all integer values.
- The inverse of *Z* exists.
- The inverse of *Z* likewise consists of all integer values.

With *Z* satisfying this condition, the float ambiguity is subjected to a variable transformation.

(4) $$z=Z^{T}a,\;Q_{zz}=Z^{T}Q_{aa}Z$$

In this situation, the integer least squares method is used to search for the optimal solution that minimizes the evaluation function corresponding to the variable transformation.

(5) $$C\left(z_{fix}\right)=argmin{\left(z_{fix}-z_{float}\right)}^{T}Q_{zz}{}^{-1}\left(z_{fix}-z_{float}\right)$$

Once the search for the optimal solution *z* is completed, the inverse z-transform is applied to convert it to the actual position parameters.

(6) $$a=Z^{-T}z$$

(7) $$b_{fix}=b_{float}-Q_{ba}Q_{aa}{}^{-1}\left(a_{float}-a_{fix}\right)$$

In this manner, cm-class position estimation is performed in RTK.

Further, a test is performed to determine whether the fix solution obtained using the evaluation function of the LAMBDA method is plausible. This test is referred to as the ratio-test [37,38,39]. The ratio-test is evaluated by the ratio of the evaluation functions of the first and second solutions obtained by the search of the LAMBDA method and is defined by the following equation.

(8) $$If\;\;Ratio=\frac{{\left(z_{fix.No2}-z_{float}\right)}^{T}Q_{zz}{}^{-1}\left(z_{fix.No2}-z_{float}\right)}{{\left(z_{fix.No1}-z_{float}\right)}^{T}Q_{zz}{}^{-1}\left(z_{fix.No1}-z_{float}\right)}\;\;\{\begin{array}{c} \;\;\geq threshold\;use\;z_{fix.No1}\\ <threshold\;use\;z_{float} \end{array}$$

If the value obtained in Equation (8) is above a certain threshold, the fix solution is considered to be an accurate search solution and is converted into a position result using Equation (7). In contrast, if the solution does not pass the test, the search solution is considered to be inaccurate, and the float solution is converted to the position result. Thus, the ratio-test makes a judgment based on whether the float ambiguity is close in value to the first solution. This is how the fix solution was tested in the past, and the method is also employed in RTKLIB.

**Figure 1:**
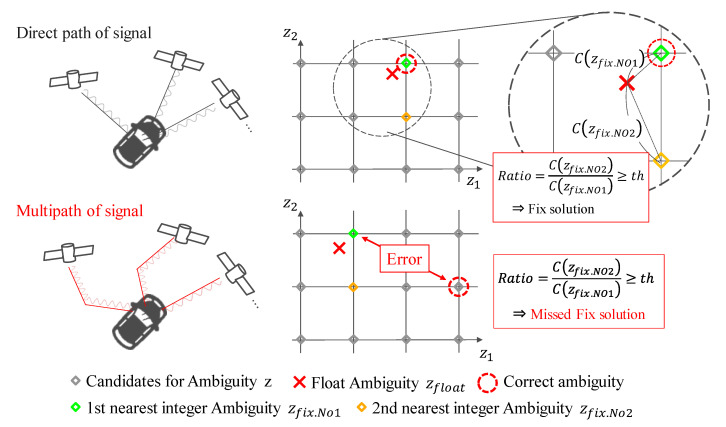
Description of ratio-test and its failure; In the red boxes *th* stands for threshold for Ratio (Equation (8)).

However, when multipath noise occurs, the ratio-test may break down. If the ratio-test fails, a fix solution will be generated with an error. Figure 1 illustrates this situation. The following is a description of the mechanism behind the occurrence of the fixed solution with the error.

Multipath noise due to high obstructions affects the pseudo-range and the carrier phase. Due to multipath noise, the observed noise becomes non-normally distributed. Because the Kalman filter assumes a normal distribution, it may fail to estimate the float ambiguity. Even the failed float ambiguity is searched by the LAMBDA method. Typically, the LAMBDA method fails in the search or is considered unsuccessful by the ratio-test. However, there are cases where the LAMBDA method searches for the first solution near the failed float solution, even though it is far from the true value. In this case, the ratio-test considers it as a pass and outputs it as a fix solution. In reality, it is the fix solution that is far from the true value, and it contains a large error. Under these conditions, the ratio-test fails and outputs the fix solution with a large error. Therefore, we believe that it is necessary to determine the fix solution in a way different from that of the ratio-test.

## 3. Proposed Method

### 3.1. A method for Determining the Reliability of Fix Solutions Using Height Trajectories

**Figure 2:**
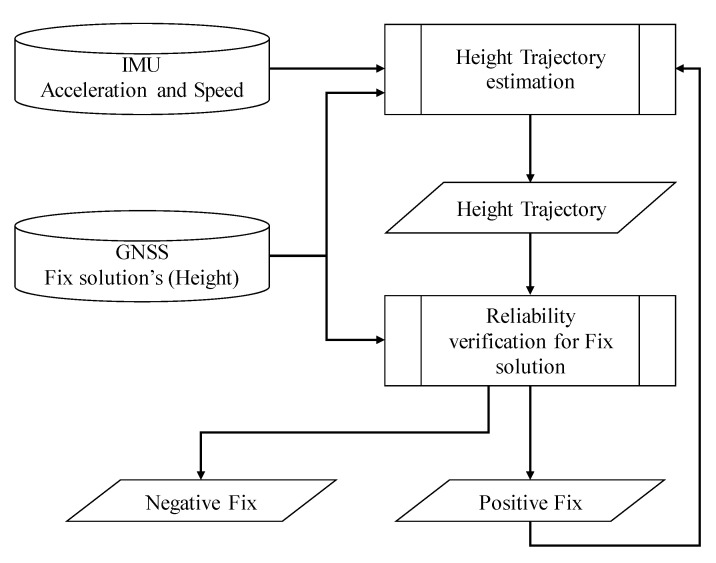
Schematic of confidence determination method.

The ratio-test, which has been applied in the conventional method, may fail in urban areas. We propose a novel method to re-estimate its reliability using the height variation as a restraint. In this paper, we refer to the variation of height as “height trajectory”. Figure 2 shows the outline of the proposed method. There are two major components of the proposed method. The first part is the estimation of the height trajectory. The height trajectory is estimated by the pitch angle and vehicle speed considering the vehicle motion. The second part is to estimate the reliability by comparing the height trajectory with the fix solution.

The determination of the reliability targets the fix solution with an error of 0.3 m or less in the horizontal plane. In the conventional method [34], the position is estimated by constraining the traveling trajectory on a plane. In this method [34], GNSS positioning solutions, with errors that do not match the shape of the trajectory, are detected in the process. However, the flatness accuracy of the trajectory is 0.5 m per 100 m, which is not sufficiently accurate to make a decision. Further, if the fix solution is output over a long distance with the offset, the trajectory shapes may match. In such a case, this would result in a wrong decision. We are able to estimate the height trajectory with an accuracy of 0.3 m per 100 m. In contrast, GNSS is prone to errors in the height direction due to factors in the geometric arrangement of satellites. The height accuracy is approximately three times worse than the horizontal accuracy. Therefore, the proposed method determines the reliability of GNSS with a larger error from the height trajectory that can be estimated with high accuracy. By taking advantage of this feature, the proposed method determines the fix solution with high reliability, and it is defined in Table 1.

### 3.2. Estimation of Height Trajectory

**Figure 3:**
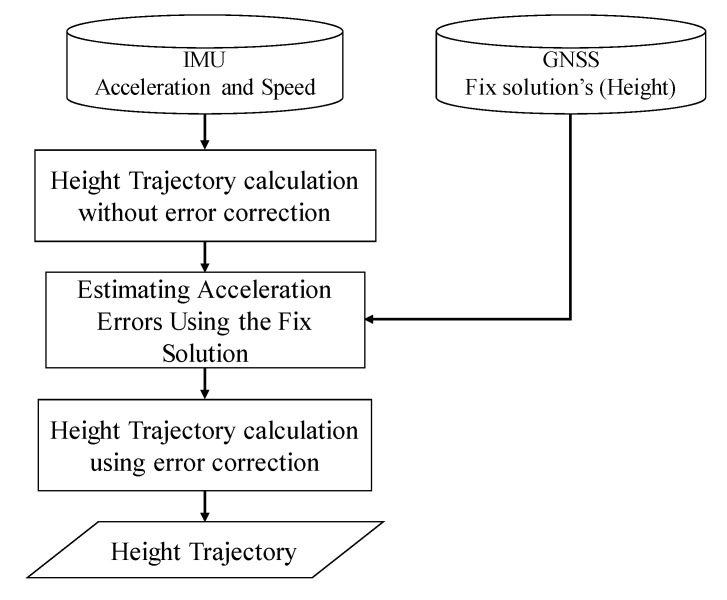
Diagram of height trajectory estimation algorithm.

This section describes the estimation of the height trajectory in the proposed method. Figure 3 shows the flowchart of the estimation. The height trajectory *H* is estimated using the vehicle speed *V* and pitch angle *θ*, according to the following Equation:

(9) $$H=H_{0}+\int Vsin\theta dt$$

**Figure 4:**
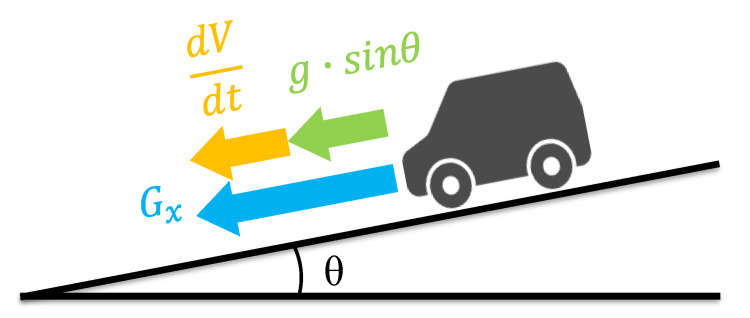
Relationship between laws of motion of a car on a slope.

In Equation (9), an initial value of *H*_0_ is set for the height, as the relative trajectory is calculated from the vehicle speed and pitch angle. Because the pitch angle is not output from the IMU, it must be estimated. We estimate the pitch angle using the acceleration of the IMU and the vehicle speed. When the vehicle motion is considered, the relationship is as shown in Figure 4 and can be expressed by the following equation.

(10) $$G_{x}=gsin\theta +\frac{dV}{dt}$$

where *G_x_* is the acceleration in the longitudinal direction, *g* is the acceleration due to gravity, and *dV/dt* is the acceleration due to change in velocity upon the car driver’s choice. In Equation (10), the pitch angle can be estimated by solving for the pitch angle.

If we assume that low-cost IMUs are used, we must take into account the error caused by the bias, etc., of the IMU. We define an error model for the acceleration of the IMU as follows:

(11) $$G_{x}{}^{true}=sf\;\cdot \;G_{x}{}^{imu}+\delta G_{x}{}^{imu}$$

where *sf* is the scale factor of acceleration, and *δG_x_* is the offset due to bias. If the error model in Equation (11) is not considered, the estimation performance by the low-cost IMU is reduced significantly. In summary, using the vehicle speed and the acceleration of the low-cost IMU, the height trajectory is obtained from Equations (9)–(11) as follows:

(12) $$H_{imu}=H_{0}+\int V\left(\frac{sf\;\cdot \;G_{x}{}^{imu}+\delta G_{x}{}^{imu}-\;\frac{dV}{dt}}{g}\right)dt$$

Therefore, it is necessary to accurately estimate the bias and scale factor, which represent errors in the acceleration, to accurately estimate the height trajectory.

**Figure 5:**
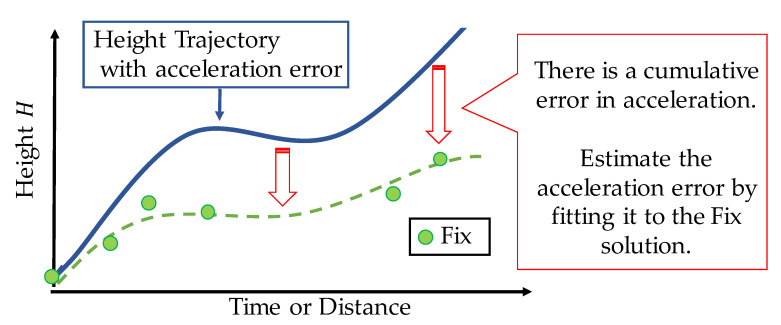
Difference in fix solution caused by acceleration error with respect to time (or distance).

In the proposed method, the error in the acceleration is estimated using the fix solution. A schematic of the acceleration error estimation is shown in Figure 5. First, the height trajectory $H_{imu}^{-}$ is estimated using Equation (12) without estimating the acceleration error. As shown in Figure 5, the difference in the fix solution naturally increases with time (or distance) due to the acceleration error. We estimate the acceleration error, such that the height trajectory $H_{imu}^{-}$ is fitted to the shape of the fix solution. Specifically, we estimate the bias and scale factor of the acceleration, such that the evaluation function defined below is minimized.

(13) $$C\left(sf,\delta G_{x}{}^{imu}\right)=argmin\overset{N}{\underset{t=t_{0}}{\sum }}{\left(H_{imu}^{-}-H_{fix}\right)}^{2}$$

The initial value of the height trajectory *H*_0_ in Equation (13) represents the first fix solution in the interval to be optimized. After estimating the error in acceleration using Equations (13) and (12) it is employed to estimate the exact height trajectory $H_{imu}^{+}$. The error in the acceleration can be regarded as constant within a certain amount of time. Therefore, in Equation (13), time series data over a long period of time (several hundred meters to several kilometers) are used for optimization. By using the long time series data, the acceleration error is plausibly estimated. This estimation procedure enables accurate estimation of the height trajectory.

### 3.3. Fix Solution Verification

**Figure 6:**
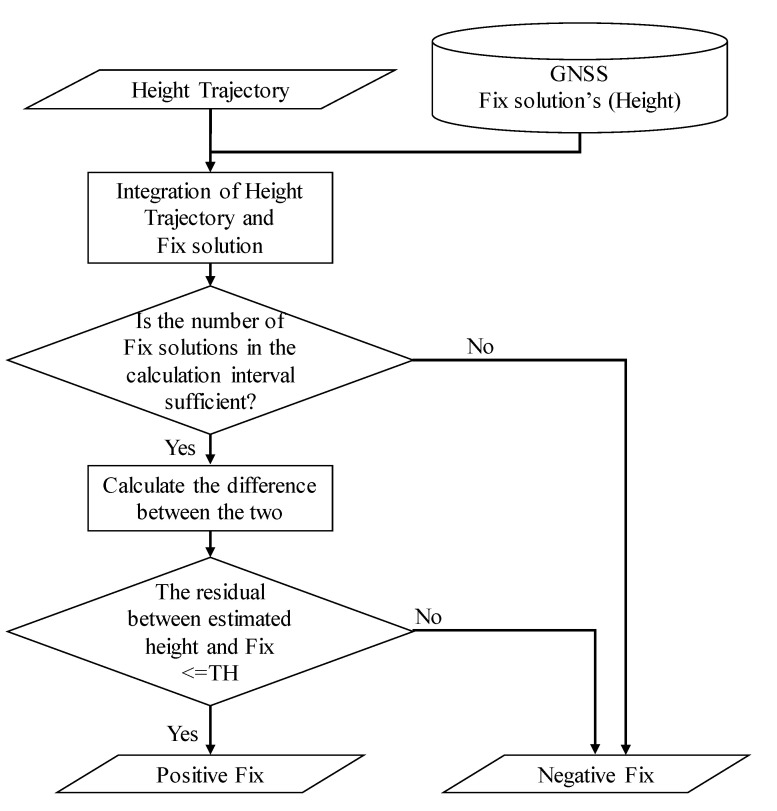
Flowchart for determining the reliability of the fix solution.

We describe the re-determination of the fix solution reliability. The flowchart of the estimation is shown in Figure 6. Primarily, the trajectory of the height is fitted to the fix solution. For fitting, we integrate the height trajectory and the fix solution such that the sum of squares is minimized. Hence, the initial value of the height trajectory, *H*_0_, is estimated such that it is minimized by the following equation:

(14) $$C\left(H_{0}\right)=argmin\overset{N}{\underset{t=t_{0}}{\sum }}{\left(H_{imu}^{+}-H_{fix}\right)}^{2}$$

**Figure 7:**
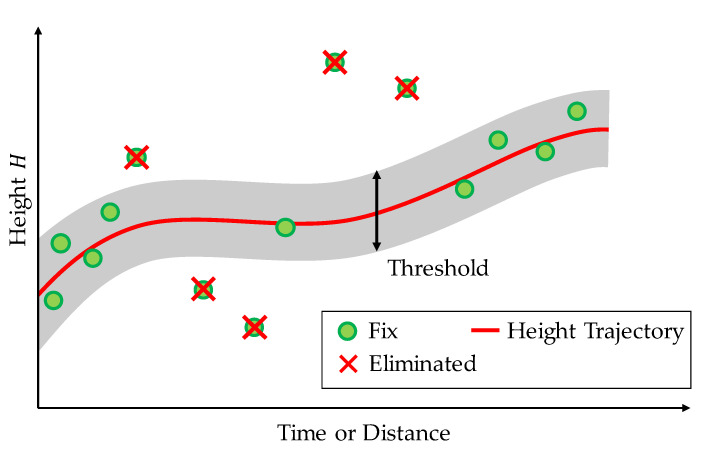
Fitting height trajectory to fix solution to determine confidence level.

At this time, as shown in Figure 7, there may be a fix solution whose shape does not match the height trajectory. If the distance from the height trajectory is above a certain threshold (0.3 m in this study), the fix solution is assumed to have an error. Because the fix solution is considered to have errors, we assign it as negative fix, as the reliability is low. In contrast, if the error is within the threshold, the solution is classified as a positive fix, as the reliability is high. In this manner, the proposed method determines the reliability of the fix solution. Further, we use 100 m for determining the reliability, instead of several hundreds of meters or kilometers like estimating acceleration error. This is because the data of a longer distance will cause more degradation of judgment accuracy, considering the case of residual errors in acceleration and vehicle speed. If the number of fix solutions obtained in a 100 m interval is low, we assume that the reliability of the estimate will decrease, and we consider it as negative fix.

**Figure 8:**
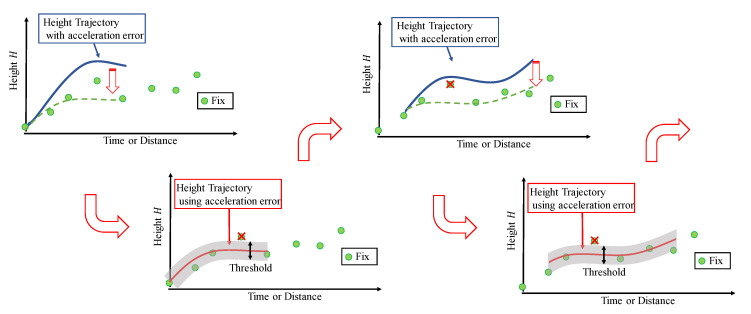
Schematic of proposed algorithm for sequential decision making.

However, the proposed method suffers from causal issues, such as “Which came first, the chicken or the egg?”. The estimation of acceleration error requires the fix solution with high reliability. However, to determine the reliability of the fix solution, it is assumed that the acceleration error is accurately estimated. This implies that the method is subject to conflict. Therefore, we solve this problem by iterating these estimates. Figure 8 and the iterative estimation method are shown below.

1. Estimate the acceleration error without determining the fix solution.
2. Determine the fix solution using the estimated acceleration error.
3. Update the time.
4. Estimate the acceleration error using the judged fix solution.
5. Repeat these steps.

By repeating the procedure in this manner, we can improve the estimation accuracy.

## 4. Evaluation Test

### 4.1. Summary of Evaluation Test Conditions

**Figure 9:**
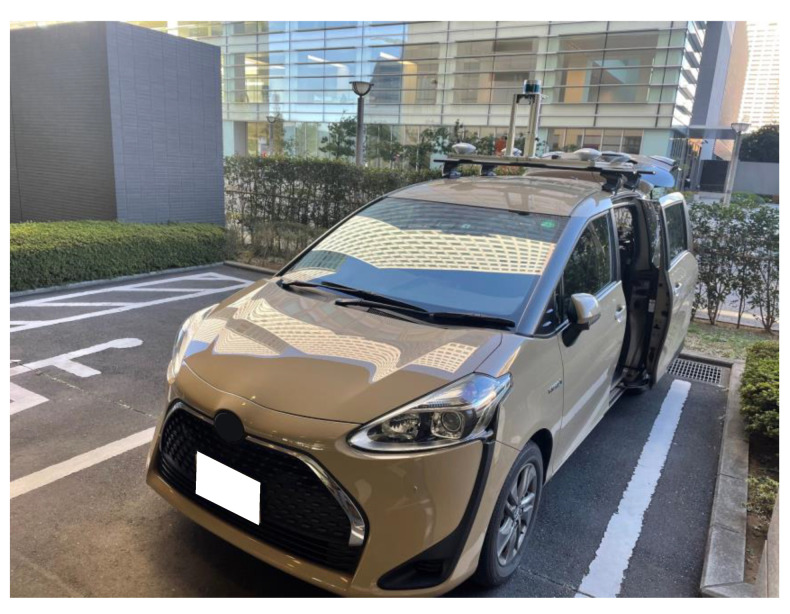
Vehicle used for evaluation tests.

To verify the effectiveness of the proposed method, an evaluation test was conducted in an urban area of Tokyo. Two evaluation routes were prepared for the test. The first route (Route A) represents a standard urban area with numerous buildings and viaducts. The second route (Route B) is a dense urban area surrounded by high-rise buildings. The list of sensors used in the evaluation test is shown in Table 2. The sensor configurations are low-cost. The GNSS receiver is a U-blox F9P mounted on the vehicle, and the acquisition period is set to 5 Hz. The satellite systems GPS, QZSS, and BeiDou are used to calculate the position of the F9P. The vehicle speed was obtained from the CAN bus of the vehicle. The MEMS-IMU is made by Tamagawa, and it is capable of measuring six axes with an acquisition period of 50 Hz. The position measured by POSLV220, a high-precision position measurement system manufactured by Applanix, is used for the reference device. The POSLV system comprises kinematic positioning, a high-precision gyro used in aircraft, and a high-resolution vehicle speed indicator (DMI). By integrating these measurements in a post-processing step, the system can estimate the position of a vehicle with an accuracy of less than 0.3 m even in an urban environment. The appearance of the vehicle equipped with the system is shown in Figure 9.

### 4.2. Test in Urban Area (Route A)

**Figure 10:**
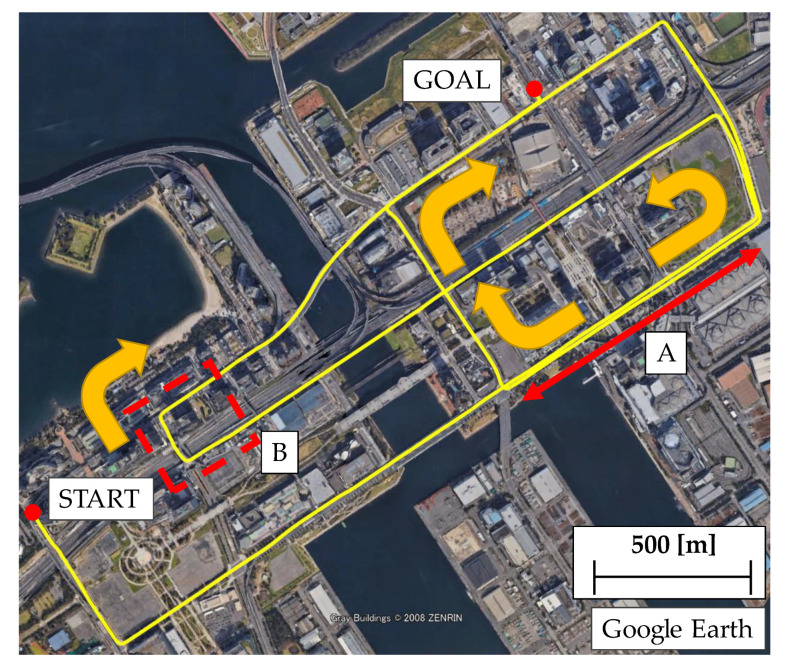
Test route A in urban environment.

The results of evaluation tests under standard urban conditions are presented. The driving route used in the evaluation test is shown in Figure 10. This route is approximately 12.5 km long. Each of the locations shown in Figure 10 is a place where GNSS positioning results are likely to deteriorate. Part A is a route that runs under the elevated railroad tracks, where the number of available satellites is likely to decrease, and signal disconnection is expected to occur. In part B, the signal is expected to be reflected and diffracted in situations where buildings are lined up, and the signal passes under a pedestrian bridge. In such a situation, it is not only difficult to obtain a fix solution, but it is also likely to generate one with errors.

**Figure 11:**
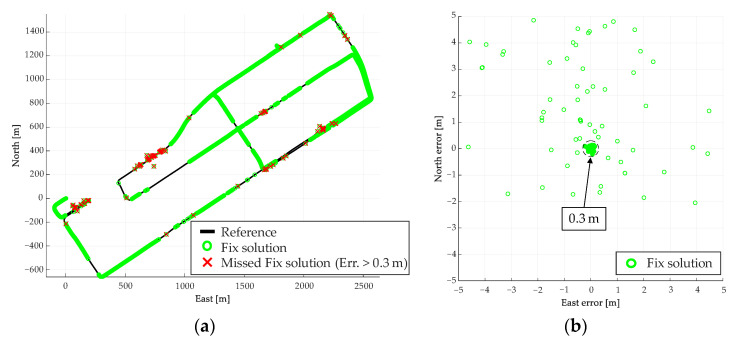
Results of real time kinematic (RTK) performed on test route A. (**a**) Fix solution for the entire route, (**b**) Error distribution of fix solution.

The results of the fix solution using RTK obtained for this route are shown in Figure 11. Figure 11a shows the results of the overall fix solution, and 11b shows the error distribution of the fix solution. In Figure 11a, the areas with errors of 0.3 m or more are highlighted in the obtained fix solutions. Numerous fix solutions with errors were obtained on the routes A and B shown in Figure 11a. In this result, the wrong fix solution accounts for 2.1% of the obtained fix solutions.

**Figure 12:**
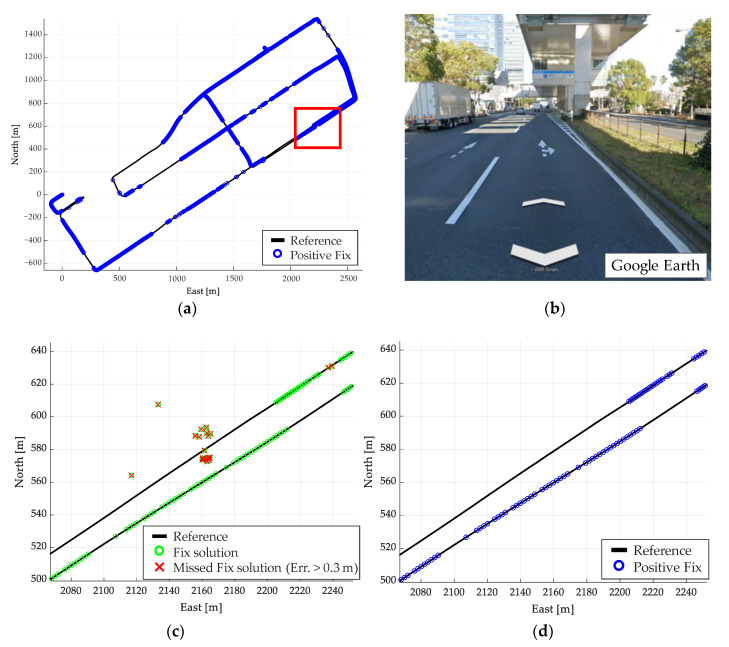
Results of reliable assessments using Route A evaluation method. (**a**) Positive fix for the entire route. (**b**) Environment in the red box. (**c**) Fix solution in the red box. (**d**) Positive fix in the red box.

**Figure 13:**
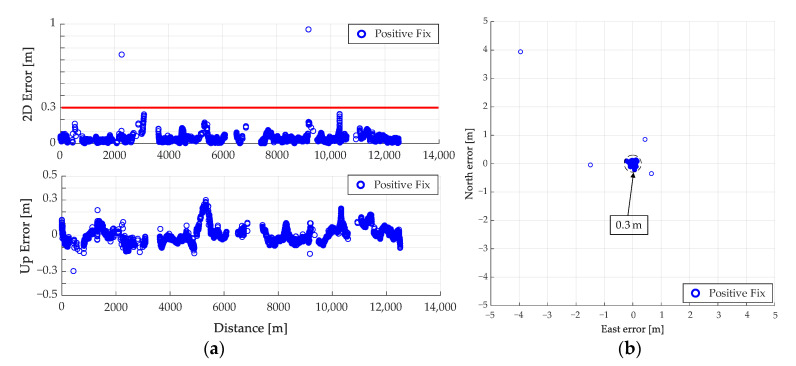
Error of positive fix by proposed method for Route A. (**a**) Positive fix plane and height error, (**b**) Error distribution of positive fix.

The overall result of the judgment using the proposed method is shown in Figure 12. Figure 13a shows the plane and height errors, and Figure 13b shows the error distribution in the plane. Table 3 shows the mean, maximum, standard deviation (SD), and root mean square (RMS) of the plane (2D) and height errors. The total number of the fix solutions determined by the proposed method is summarized in Table 4. By comparison of Figure 11 and Figure 12, most of the conventionally fix solutions have errors of 0.3 m or above, while the positive fix solutions by the proposed method are mostly within 0.3 m. From Table 3, the RMS of the plane decreases from 4.16 to 0.17 m, which means that the fix solution can be determined with high reliability. Table 4 shows that the positive fix of 99.9% (6369/6373) is within the error of 0.3 m with the proposed method. Therefore, the proposed method can determine the reliability more accurately than the conventional one.

Further, there are four positive fixes with large errors in Table 4. This is attributed to a large error in the horizontal plane, even though there is no error in the height direction. In such a case, the reliability can be determined by combining it with the determination by planes in the method [34].

### 4.3. Test in Dense Urban Area (Route B)

**Figure 14:**
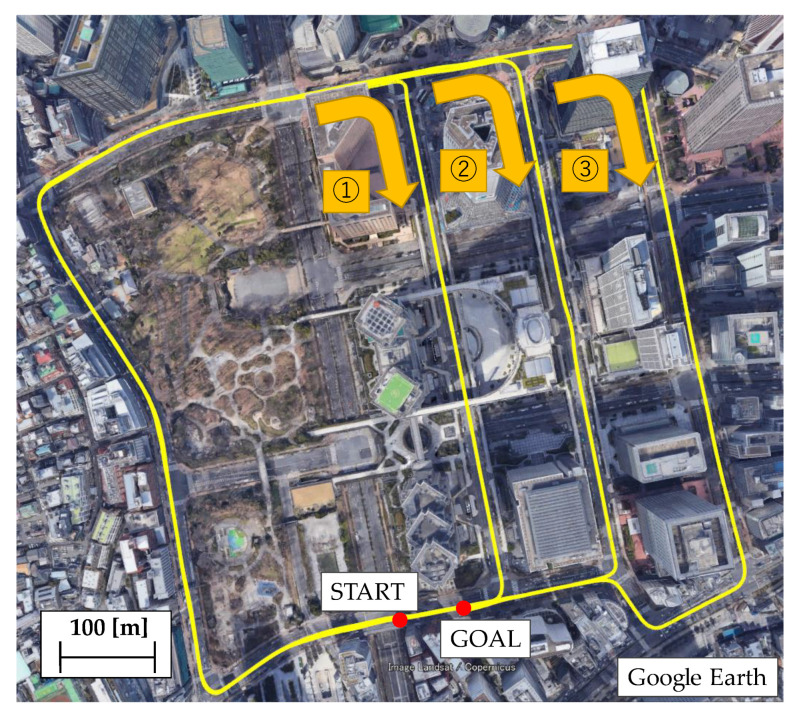
Test route B in a dense urban environment.

**Figure 15:**
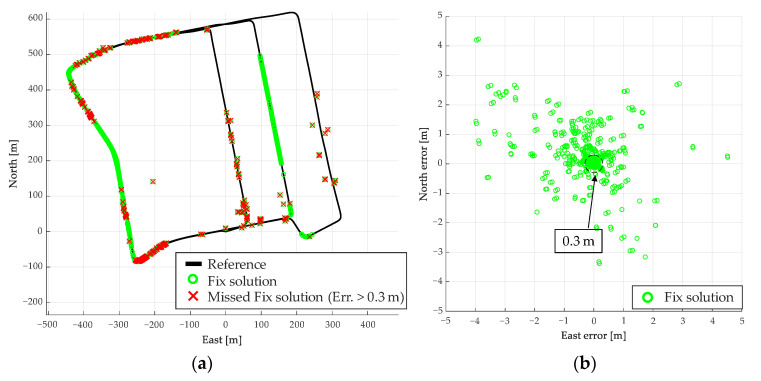
Results of RTK performed on test route B. (**a**) Fix solution for the entire route (**b**) Error distribution of fix solution.

The results of the evaluation test in a dense urban environment with many high-rise buildings in the city center are described. Figure 14 shows the test route used for the evaluation (6.6 km). This evaluation route is lined with buildings over 100 m high. Consequently, multipath is likely to occur, and accurate satellite positioning is difficult. The RTK results for this route are shown in Figure 15.

Similarly to the test for Route A, Figure 15a shows the results of the overall fix solution, and 15b shows the error distribution of the fix solution. From Figure 15, we see that there are fewer fix solutions obtained than for the results of Route A, and more fix solutions with large errors. The fix solutions with errors account for 8.6% of the obtained fix solutions, which is below that of Route A.

**Figure 16:**
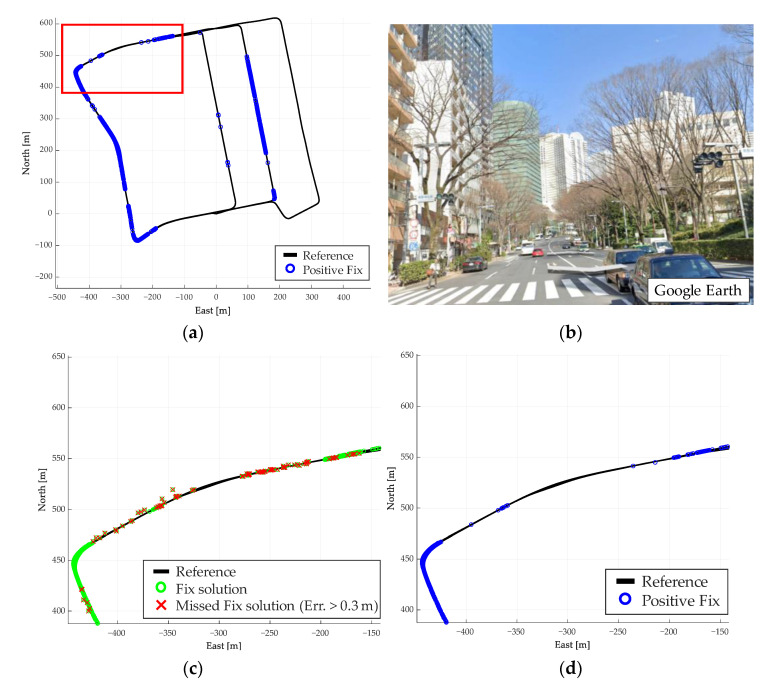
Results of reliability determination using Route B evaluation method. (**a**) Positive fix for the entire route, (**b**) Environment in the red box, (**c**) Fix solution in the red box, (**d**) Positive fix in the red box.

**Figure 17:**
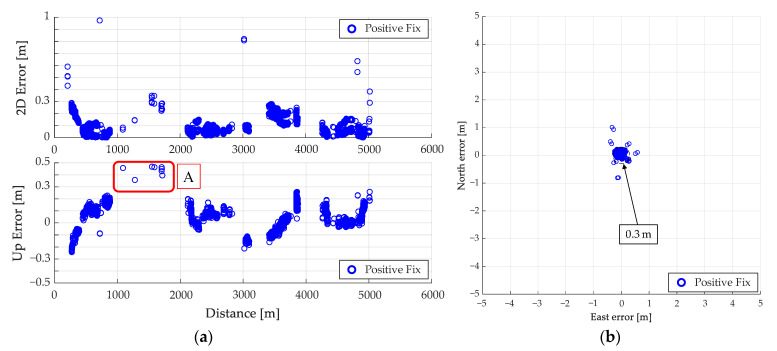
Error of positive fix by proposed method for Route B. (**a**) Positive fix plane and height error, (**b**) Error distribution of positive fix.

We apply and evaluate the proposed method in this course as well. Figure 16 shows the results of the judgment using the proposed method. The plane and height errors are shown in Figure 17a, and the error distribution in the plane is shown in Figure 17b. As in Route A, Table 5 shows the mean, maximum, SD, and RMS of the plane (2D) and height errors. The total number of fix solutions determined by the proposed method is summarized in Table 6.

Figure 16 shows that several fix solutions are determined as negative fixes by the proposed method. Therefore, the reliability determination operates accurately even in a multipath environment lined with buildings. As shown in Figure 17, most of the fix solutions, which are considered positive fixes, are within 0.3 m error. The RMS of the plane in Table 5 likewise shows that the proposed method reduces the error compared to the conventional fix method. Table 6 shows that the percentage of the error within 0.3 m is 99.7% (5370/5384) for the positive fix. Therefore, it is confirmed that the proposed method can be used to determine the reliability with high accuracy in this evaluation test as well.

However, compared to the evaluation test A, the accuracy of the reliability determination has deteriorated. In part A of Figure 17a, the correct judgment is not made, even when the height error is 0.3 m or more. This is thought to be due to the fact that the number of fix obtained is small, and therefore, the error in acceleration cannot be estimated correctly, resulting in an error in the pitch angle. An error in the pitch angle leads to an error in the relative height trajectory, and thus an accurate determination is impossible. In this case, it is necessary to estimate the bias and pitch angle of the accelerometer without using the fix solution. Therefore, by improving the fix solution itself and the accuracy of the height trajectory, a highly accurate reliability determination is expected, even in urban environments.

## 5. Conclusions

Autonomous driving support systems and self-driving cars require highly accurate and reliable vehicle positions. The fix solution of kinematic positioning is often used as a factor to show the reliability of GNSS positioning results. Most fix solutions are determined by the combination of the LAMBDA method and ratio-test. However, fix solutions with large errors are typically generated in urban areas. Therefore, in this study, we propose another method to determine the reliability in place of the ratio-test. In this study, we consider two aspects. One is that the height variation of the vehicle is small and can be estimated more accurately. The other is that GNSS errors are more likely to occur in the height direction than in the plane direction. Based on these considerations, we proposed a method for detecting a reliable fix solution by using the high trajectory during driving. According to the evaluation tests, the proposed method achieved a decision accuracy of 99.9% on route A, which is an urban area. This determination resulted in a flatness accuracy (RMS) of 0.17 m, achieving the target of 0.3 m or below. In Route B, which is a dense urban area with a harsh environment, the judgment accuracy was 99.7%. Here, the RMS of the plane was 0.22 m, indicating that the confidence level was estimated with high accuracy. Because these results were determined with a higher judgment accuracy than the ratio test, the proposed method is considered to be effective.

However, we have not yet been able to completely assess the reliability. In Route B of the evaluation test, correct assessments could not be made even when the height error was 0.3 m or more. To improve these problems, it is necessary to improve the accuracy of the height trajectory and combine the assessment in horizontal directions. Further, we must solve the multipath problem of GNSS and improve the accuracy of the fix solution.

## Figures and Tables

**Table 1:** Status naming definition of judgment by the proposed method.

Proposal Method	High Reliability	Low Reliability
Status Name	Positive Fix	Negative Fix

**Table 2:** List of equipment used in experiment.

Equipment	Manufacturer	Model
GNSS Antennas	Aero	AT1645-540T
GNSS receiver	U-blox	F9P
IMU	Tamagawa	AU7684
Speed	Toyota Sienta	CAN
Reference	Applanix	POSLV220

**Table 3:** List of errors from evaluation tests for Route A.

		Error Max (m)	Error Mean (m)	Error SD (m)	Error RMS (m)
Fix	2D	78.5	0.07	2.08	4.16
	Height	186.6	0.64	6.77	6.79
Positive Fix	2D	5.58	0.01	0.08	0.17
	Height	0.30	0.05	0.06	0.06

**Table 4:** Number of confidence levels determined by Route A evaluation tests.

Number	Conventional Fix	Proposal Positive Fix	Proposal Negative Fix
Error < 0.3 m	6372	6369	3
Error > 0.3 m	134	4	131

**Table 5:** List of errors from evaluation tests for Route B.

		Error Max (m)	Error Mean (m)	Error SD (m)	Error RMS (m)
fix	2D	390.7	0.37	7.38	14.8
	Height	808.7	0.85	13.9	13.9
Positive Fix	2D	1.07	0.05	0.10	0.22
	Height	0.73	0.11	0.10	0.13

**Table 6:** Number of confidence levels determined by Route B evaluation tests.

Number	Conventional Fix	Proposal Positive Fix	Proposal Negative Fix
Error < 0.3 m	6577	5370	1207
Error > 0.3 m	619	14	605
